# TNF-Alpha Promotes an Inflammatory Mammary Microenvironment That Favors Macrophage and Epithelial Migration in a CCL2- and Mitochondrial-ROS-Dependent Manner

**DOI:** 10.3390/antiox12040813

**Published:** 2023-03-27

**Authors:** María Jesús Vera, Francisco Guajardo, Felix A. Urra, Nicolás Tobar, Jorge Martínez

**Affiliations:** 1 Molecular and Cell Biology Laboratory, INTA, University of Chile, Santiago 7830490, Chile; 2 MIBI: Interdisciplinary Group on Mitochondrial Targeting and Bioenergetics, Santiago 8380453, Chile; 3 Metabolic Plasticity and Bioenergetics Laboratory, Program of Clinical and Molecular Pharmacology, Institute of Biomedical Sciences (ICBM), Faculty of Medicine, University of Chile, Santiago 8380453, Chile; 4 Network for Snake Venom Research and Drug Discovery, Santiago 8380453, Chile

**Keywords:** migration, monocytes, breast cancer, OXPHOS, adipocytes

## Abstract

The influence of an inflammatory microenvironment on tumorigenesis has been widely accepted. Systemic conditions that favor the onset of an inflammatory landscape predispose the progression of breast cancer. Under obesity conditions, the endocrine function of adipose tissue is one of the main determinants of the production of local and systemic inflammatory mediators. Although these mediators can stimulate tumorigenesis and recruit inflammatory cells, as macrophages, the mechanism involved remains poorly understood. In the present work, we describe that the TNFα treatment of mammary preadipocytes from human normal patients blocks adipose differentiation and promotes the generation of pro-inflammatory soluble factors. The latter stimulate the mobilization of THP-1 monocytes and MCF-7 epithelial cancer cells in an MCP1/CCL2- and mitochondrial-ROS-dependent manner. Together, these results reaffirm the contribution of an inflammatory microenvironment and mtROS in the progression of breast cancer.

## 1. Introduction

A chronic inflammatory state can constitute a functional tumoral microenvironment that, in breast cancers, is present before the establishment of the malignant state [1]. Since this inflammatory niche promotes stem-like properties in breast cancer cells, increasing the propensity to form tumors [2] and chemoresistance [3], understanding the mechanisms that trigger it can contribute to better therapeutic strategies for breast cancer.

Obesity is an independent risk and prognosis factor for breast cancer [4]. This correlation has been attributed to the obesity-dependent endocrine function of obese adipose tissue, which is capable of producing pro-inflammatory cytokines, such as Interleukin 6 (IL-6), Interleukin 8 (IL-8), leptin, and tumor necrosis factor-alpha (TNFα), that stimulate the monocyte/macrophage recruitment to the tumor microenvironment (TME) [1]. Notably, the chronic, endogenous low production of TNFα, which is one of the most relevant mediators secreted by the obese adipose tissue, promotes the mammary carcinogenic process [5].

According to the standard classification, the mammary adipose compartment corresponds to white (similar to subcutaneous) fat [6]. Despite this, under a systemic view, its functionality also depends on the equilibrium and secretory activity of the whole adipose mass. Therefore, it is affected by pathological conditions such as obesity. Obese women are at higher risk of breast cancer when compared to non-obese women. In this context, increased levels of estrogens due to the excessive aromatization activity of the adipose tissue, the overexpression of pro-inflammatory cytokines, and insulin resistance, among other factors, contribute to the development of breast cancer in obese women. All these factors, operating through different mechanisms, modulate mammary adipocyte function and thereby affect the capacity to control breast development [7]. A possible role of brown adipose tissue (which is much less abundant than the white counterpart) in cancer has been hypothesized; however, a specific role in breast cancer has not been recognized yet [8].

The mammary stroma of breast tumors experiences the suppression of preadipocyte differentiation and the induction of adipocyte dedifferentiation, generating a more fibrotic environment that facilitates cancer cell motility [9]. A high stromal collagen density produces a threefold increase in mouse mammary tumor numbers with a more invasive phenotype, producing greater local invasion and metastasis [10].

In humans, mammographic studies have identified, in normal breasts, areas with a low adipocyte content and a high extracellular matrix (ECM) and stromal cell abundance which appears denser in the image [11]. Epidemiological studies have shown that the involvement of 60% or more of the breast tissue with mammographically dense areas confers a three to five-fold increased relative risk for breast cancer [12]. Considering that desmoplastic lesions are present in the absence of tumor cells, this feature constitutes a preexisting condition that favors breast cancer development [13].

TNFα is consistently high in the breast tumor microenvironment, which exerts a variety of functions that affect cell malignancy and metabolism [14]. More recently, it has been demonstrated that TNFα regulates the survival and proliferation of breast cells, modulating mitochondrial metabolism and affecting respiratory chain supercomplex organization and function [15].

In the present work, we focused our research on TNFα action in stromal cells, which are the more abundant phenotype in breast tumors. For this purpose, we used human breast preadipocytes in cultures derived from healthy patients treated with TNFα and analyzed the effect of this factor on adipose differentiation. We also studied the main properties of MCP1/CCL2, a stromally secreted factor generated by these treated cells, in (i) the capacity of soluble factors to stimulate the recruitment of monocytes, (ii) the potential for these factors to stimulate epithelial migration, and (iii) the capacity of these stromal-soluble factors to generate an oxidative environment that favors epithelial migration. Our results suggested that human mammary preadipocytes treated with TNFα display a blockade of its adipose differentiation, and that either the MCP1/CCL2 or ROS produced by these TNFα-treated cells constitute a probable mediator of the establishment of an inflammatory environment that promotes monocyte recruitment and epithelial migration.

## 2. Materials and Methods

### 2.1. Isolation and Culture of Human Mammary Stromal Cells

Human mammary fat tissue was obtained from nine healthy females (without obesity, diabetes, or hypertension) undergoing elective breast cosmetic surgery (mammoplasty). The patients’ donors had a mean ± SD age of 32.1 ± 4.2 years and a body mass index of 22.5 ± 2.8 kg/m^2^. The donors signed an informed consent form, and the protocol was approved by the Institutional Review Board of INTA, University of Chile. The collected tissue was washed with phosphate-buffered saline (PBS) solution, cleaned, and minced into small pieces (2–3 mm^2^). The isolation of human mammary stromal cells (primary preadipocytes) was based on the explant culture method described previously [16]. Tissue and tissue-derived cells were cultured in DMEM/F12 supplemented with 10% FBS, antibiotics (penicillin/streptomycin), and an antifungal (amphotericin B) at 37 °C in a controlled atmosphere incubator. After passages 2–3, the resulting cells were harvested and seeded to expose to the different experimental conditions.

### 2.2. Primary Cell Culture and Adipogenesis

Cells were obtained and grown in the above-mentioned conditions. At passages 3–4, mammary cells were seeded at 10.5 × 10^3^ cells/cm^2^ and exposed to the differentiation cocktail (DMEM: F12 supplemented with 0.28 UI/mL insulin, 0.5 mM methyl-isobutyl xanthine, 1 mM dexamethasone, and 1 µM rosiglitazone) for 14 days [17]. The culture medium was replaced every 3 days, and the mature adipocytes’ phenotype was evaluated after exposure to the adipogenic cocktail for 7 or 14 days by the adipocyte marker expression (qPCR) and triglyceride accumulation (lipid droplets), respectively.

### 2.3. Cell Lines and Chemicals

A MCF-7 human breast cancer cell line was purchased from ATCC (Manassas, VA, USA), cultured in DMEM/F12 supplemented with 10% FBS, and maintained in a humidified atmosphere of 37 °C and 5% CO_2_. THP-1 monocyte cells were also purchased from ATCC and were cultured in RPMI medium supplemented with 10% FBS under the same conditions as those described above. Tumor necrosis factor alpha (TNFα) and MCP1/CCL2 were obtained from the R&D system (Minneapolis, MN, USA). The MCP1/CCL2 protein was evaluated in media conditioned by preadipocytes and differentiated adipocytes (treated or not treated with TNFα), using an ELISA method (R&D system, Minneapolis, MN, USA catalog number DY279-05) according to the manufacturer’s instructions.

### 2.4. Preparation of Conditioned Media

A stromal conditioned media (CM) was prepared through two procedures: (i) from confluent cultures of preadipocytes from different donor patients, which were pretreated or not treated with 20 ng/mL of TNFα for 72 h. After this treatment, the cells were washed twice and cultured in a serum-free medium for 24 h. The media were collected and filtrated to be used. (ii) Preadipocytes from the same human donor were cultured in adipogenic media, the composition of which was as described above, for 14 days. After this treatment, the cells were treated with TNFα for 72 h and then cultured in a serum-free medium for 24 h, as above.

### 2.5. Oil Red O Staining of Cytoplasmic Triglyceride

After 14 days of adipogenic stimulus, mammary preadipocyte monolayers were washed twice with PBS and fixed with 3.7% formaldehyde for 2 min. A 0.2% Oil Red O-isopropyl alcohol solution was added to the cells for 1 h, after which the monolayers were washed several times with distilled water, and the stained cytoplasmic triglycerides were visualized and measured using the Image J software v 1.53a [17].

### 2.6. Cell Migration Assay

The MCF-7 cell migration was studied using a 6.5 mm Transwell chamber with a pore size of 8 mm (Corning, Corning, NY, USA). Cells were allowed to migrate (48 h) under the stimulus of conditioned media by preadipocytes that were pre-treated or not treated with TNFα (described in the Section 2), and 10% FBS was used as a positive control. In the case of the THP-1 cells, recruitment potential was evaluated by a similar migratory assay, using a 6.5 mm Transwell chamber (Corning, Corning, NY, USA) with a pore size of 5 mm. In some experiments, migration was also stimulated with the use of 10 ng/mL of MCP1/CCL2 as a positive control. In experiments in which migration interfered with a blocking antibody against MCP1/CCL2 (10 µg/mL, R&D system, Minneapolis, MN, USA), this molecule was added to the lower chamber during the migration assay. In the case of interfering migration with mitoTEMPO (1 µM, Sigma Aldrich, St. Louis, MO, USA), this molecule was added to preadipocytes during the 72 h period of TNFα stimulation prior to the preparation of conditioned media. After the migration period, cells that migrated across the membrane were fixed in methanol and stained with 0.2% crystal violet [18]. The migration values corresponded to the average of three independent experiments by counting 8 fields from the four pictures (×20) per chamber (two chambers per experimental condition).

### 2.7. Quantitative PCR

Total RNA was isolated from the preadipocytes and mature adipocytes with Trizol (Ambion, Carlsbad, CA, USA), according to manufacturer instructions. A reverse transcription to complementary DNA was performed with 1 µg of RNA from each sample using an M-MLV reverse transcriptase and oligo-dT (Promega, Madison, WI, USA) as a primer, according to manufacturer protocol. PPARγ, AP2, IL-1β, and CCL2 messenger RNA (mRNA) expression was assessed via real-time PCR using a Light Cycler instrument (Roche, Basel, Switzerland). The reaction was performed using 200 ng of complementary DNA and a LightCycler1 FastStart DNA Master SYBR Green I kit (Roche) with a final volume of 10 µL. All the reactions were performed in duplicate, and negative controls were included. GPDH was used for housekeeping. The primer sequences were described in Table 1.

The sequences of the primers were as follows:

### 2.8. Mitochondrial ROS (mtROS) Levels

The mtROS levels were measured using staining with MitoSOX^®^ Red probe (Invitrogen, Carlsbad, CA, USA). MCF-7 (1.5 × 10^5^ cells/mL) was seeded into 12-well plates, incubated for 24 h, washed with PBS, and treated with fresh medium or CM from preadipocytes (pre-treated or not treated with TNFα) for 24 h. Next, cells were incubated with MitoSOX Red^®^ (5 µM) for 30 min. They were then recollected and washed, and the fluorescence was detected by flow cytometry as described [19].

### 2.9. Statistical Analysis

All statistical analyses were performed using Graph Pad Prism 5.0 (GraphPad Software, San Diego, CA, USA). A statistical analysis was performed using the Friedman test, followed by Bonferroni’s multiple comparisons test, or the Kruskal–Wallis test, followed by Dunn’s multiple comparisons test, as mentioned in the legends. The data were considered statistically significant when *p* < 0.05.

## 3. Results

### 3.1. TNFα Inhibits In Vitro Adipose Differentiation in Human Mammary Pre-Adipocytes

**Figure 1:**
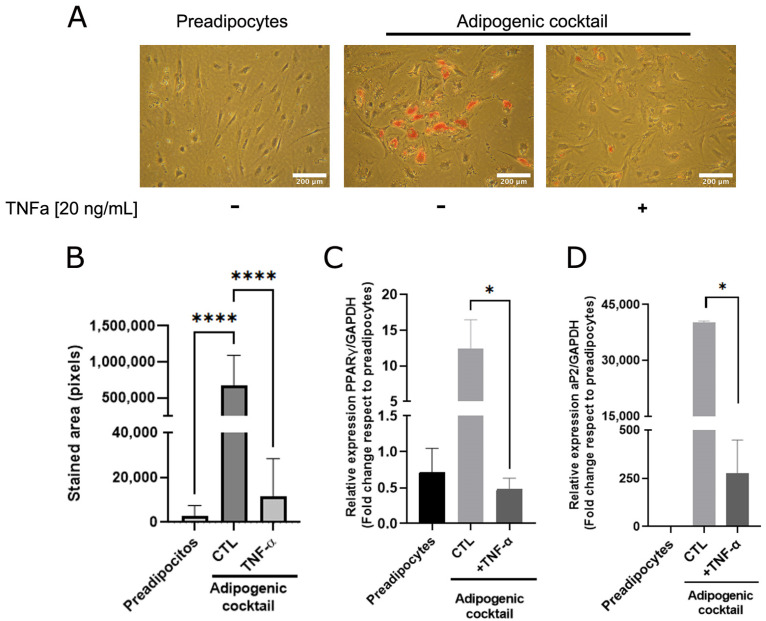
TNFα inhibits adipose differentiation in human mammary pre-adipocytes. Stromal cells derived from nine healthy donors were cultured in the presence or absence of an adipogenic cocktail, according to Section 2, and pre-treated or not treated with TNFα (20 ng/mL) as inflammatory stimuli. After 14 days, triglyceride accumulation in differentiated cells was visualized by Oil Red O. (**A**) Representative images (×10) of the nine samples tested. (**B**) Quantification of the lipid droplet area by Image J software. Bars represent the mean ± SD of nine samples assayed. Kruskal–Wallis test, followed by Dunn’s multiple comparisons, test was used to derive all *p* values. (**C**,**D**) Relative expression by qPCR of early PPARγ (7 days after stimulus) and late aP2 (14 days after stimulus) differentiations markers from preadipocytes treated or not with adipogenic cocktail and TNF**α**. Differentiation markers assay was performed with three different samples in three independent experiments. * *p* < 0.05 and **** *p* < 0.0001.

As adipose cells constitute the most abundant phenotype of mammary tissue and are essential for mammary ductal morphogenesis [20,21], we analyzed whether TNFα, a cytokine that plays a relevant role in the inflammatory state, affects adipose differentiation. A sample collection of human mammary preadipocytes derived from healthy donors was exposed to the adipogenic cocktail (see Section 2) in the absence or presence of TNFα (20 ng/mL). As Figure 1A shows, preadipocytes under adipogenic cocktail conditions exhibited an increase in lipid load, which was significantly prevented by TNFα (Figure 1A,B). To reinforce the concept that cytokine activity affects the transcriptional adipogenic machinery, the changes in the PPARγ and AP2 gene expression in human preadipocytes under differentiation was determined. As Figure 1C,D shows, TNFα inhibits the expression of these adipogenic markers, which was stimulated by the adipogenic cocktail. These results suggest that TNFα induces a terminal blockade of the adipogenic process.

### 3.2. Soluble Factors Derived from TNFα-Treated Preadipocytes Stimulate Monocyte Recruitment

**Figure 2:**
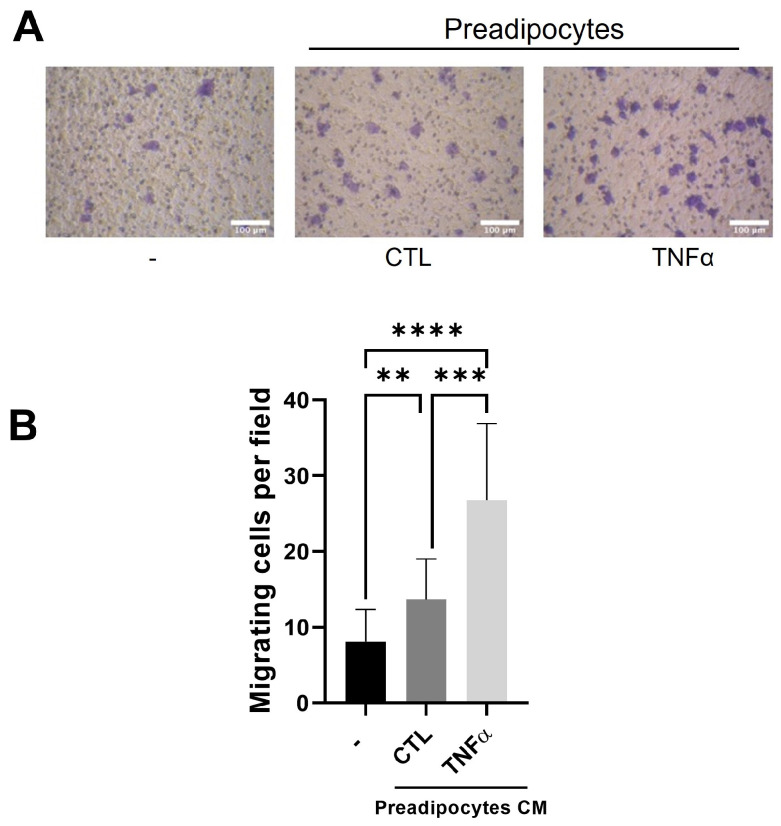
Conditioned media with TNFα-treated preadipocytes stimulate monocyte recruitment. THP-1 monocytes (1 × 10^5^ cells) were plated on the insert of a Transwell with pores 5 μm in diameter and subjected to migration against 10 ng/mL of CCL2 (CTL) or conditioned media (50%) with preadipocytes pretreated or not treated with TNFα (20 ng/mL). (**A**) Representative images (×20) of migratory cells in each condition. (**B**) Migratory cells were scored as indicated in Section 2 after a 6 h migration time. Data are shown as mean ± SD of three independent experiments performed with preadipocytes from three different human samples. Friedman test, followed by Bonferroni’s multiple comparisons test, were used to derive all *p* values. ** *p* < 0.01, *** *p* < 0.001 and **** *p* < 0.0001.

Tumor-associated macrophages (TAMs) are derived from monocytes attracted to the tumor inflammatory environment by locally produced chemotactic factors [22]. To test whether the soluble factors produced by mammary preadipocytes treated with TNFα recruit monocytes, we subject THP-1 monocytes to a migratory assay (6 h) using conditioned media (CM) derived from preadipocytes pretreated or not treated with TNFα (72 h) as a chemotactic factor. As Figure 2 shows, the THP-1 cells were significantly stimulated to migrate when the outer compartment of the Transwell contained CM from TNFα-pretreated preadipocytes.

### 3.3. TNFα Stimulates the Expression of Inflammatory Factors in Mammary Preadipocytes

**Figure 3:**
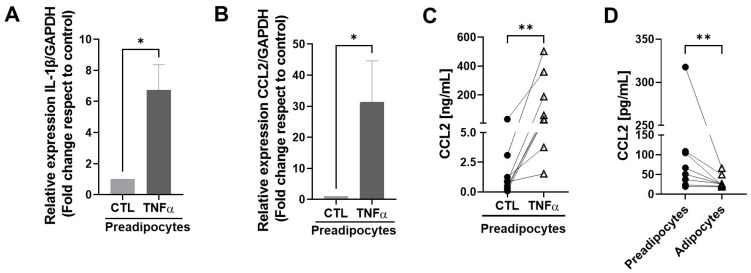
TNFα stimulates the expression of inflammatory factors in mammary preadipocytes. (**A**,**B**) Preadipocytes derived from mammary tissue from eight healthy patients were treated with TNFα (20 ng/mL) for 72 h. Relative expression of IL-1β and MCP1/CCL2 mRNAs was analyzed by qPCR. (**C**,**D**) Aliquots of conditioned media generated by preadipocytes or differentiated adipocytes treated or not treated with TNFα were evaluated for the MCP1/CCL2 abundance using an ELISA assay. Data correspond to the mean ± SD of eight different samples. Wilcoxon (**A**,**B**) and paired Wilcoxon (**C**,**D**) tests were used to deriving all *p* values. * *p* < 0.05 and ** *p* < 0.01.

To test whether TNFα-treated human mammary preadipocytes can produce factors able to maintain an inflammatory environment, we treated cultures of human preadipocytes with TNFα and evaluated the expression of IL-1β and MCP1/CCL2 mRNAs, factors that play a relevant role in the maintenance of an inflammatory status in mammary tissue [23]. Figure 3A,B show that cells treated with TNFα generated significantly more CCL2 and IL-1β mRNA copies compared with the controls. Figure 3C shows the augmented expression of MCP1/CCL2 as a protein measured by an ELISA assay in conditioned media with preadipocytes derived from eight individual samples. To compare the capacity to generate the MCP1/CCL2 protein between mammary preadipocytes and differentiated adipocytes derived from the same sample cells, we analyzed the production of this chemokine in conditioned media using eight samples in basal and post-differentiation states with an ELISA assay. As Figure 3D shows, at the basal level, the capacity of preadipocytes to produce the MCP1/CCL2 protein was significantly higher than their differentiated counterpart. Mature adipocytes also were stimulated by TNFα on CCL2 production, but in a minor proportion (almost three orders of magnitude less) compared with their preadipocytes counterpart (see Supplementary Materials).

### 3.4. MCP1/CCL2 Contained in Media Conditioned by TNFα-Treated Preadipocytes Is Responsible for Monocyte recruitment

**Figure 4:**
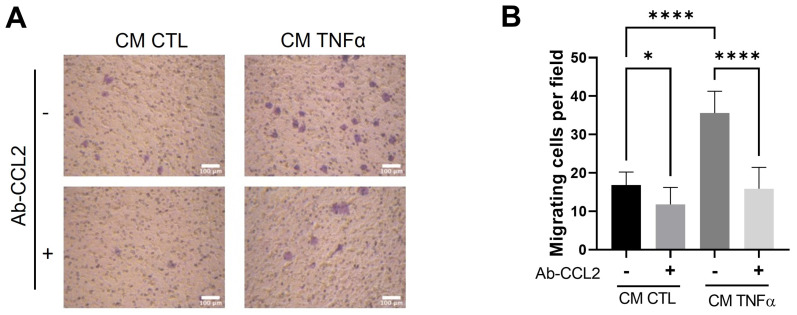
MCP1/CCL2 present in conditioned media by TNFα-treated preadipocytes stimulates monocyte recruitment. THP-1 monocytes (1 × 10^5^ cells) were subjected to a recruitment experiment following the same procedure that those in Figure 2. A group of these cells was subjected to migrate under CM stimulus (50%) from mammary preadipocytes treated or not treated with 20 ng/mL TNFα in the presence or absence of 10 µg/mL of anti-CCL2 blocking antibody (R&D system). (**A**) Representative photomicrographs (×20) of migratory cells in each condition and (**B**) bars represent mean ± SD of migrating cells per field. Friedman test, followed by Bonferroni’s multiple comparisons test, were used to derive all *p* values. * *p* < 0.05 and **** *p* < 0.0001. Data correspond to three independent experiments performed with CM from three different samples.

To test whether MCP1/CCL2, an effective contributor to the recruitment of blood monocytes into sites of inflammatory responses and tumors [24], is produced by TNFα-treated preadipocytes and participates in the monocyte mobilization, we implemented a monocyte migratory assay, as shown in Figure 2, but in which a group of cells was allowed to migrate in the presence of an anti-MCP1/CCL2-blocking antibody. As Figure 4 shows, the MPC1/CCL2-blocking antibody significantly inhibits the monocyte migration, providing evidence that MCP1/CCL2 derived from TNFα-pretreated preadipocytes is an active recruitment agent and participates in the establishment of an inflammatory mammary microenvironment.

### 3.5. Soluble Factors Derived from TNFα-Treated Preadipocytes Stimulate Cancer Epithelial Migration in an MCP1/CCL2-Dependent Manner

**Figure 5:**
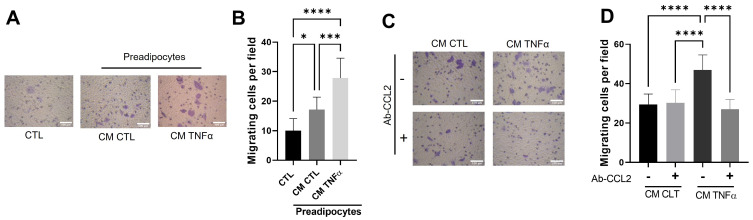
Soluble factors produced by TNFα-treated preadipocytes stimulate cancer epithelial migration in an MCP1/CCL2-dependent manner. (**A**,**B**) MCF-7 epithelial cells (2 × 10^5^) were stimulated to migrate for a 48 h period against 50% of conditioned media with preadipocytes previously treated (72 h) or not treated with TNFα (20 ng/mL), using a Transwell system. CM CTL corresponds to a media conditioned in the absence of the factor. (**C**,**D**) MCF-7 epithelial cells were subjected to migrate in the same conditions as in A but included a group of cells that migrate in the presence of an anti-CCL2 blocking antibody (10 µg/mL). Bars represent mean ± SD of migrating cells per field. Friedman test, followed by Bonferroni’s multiple comparisons test, were used to derive all *p* values. * *p* < 0.05, *** *p* < 0.001 and **** *p* < 0.0001. Data correspond to three independent experiments performed with CM from three different samples.

Once the role of conditioned media with TNFα-treated preadipocytes in the creation of an inflammatory milieu was established, we analyzed the possible effect of this media on cancer epithelial behavior. We did so by evaluating the effect of CM-derived TNFα-treated human mammary preadipocytes over 72 h on the migration of MCF-7 cells. In these experiments, CM (50%) was included in the lower chamber of the Transwells. Figure 5A shows that CM from preadipocytes pretreated with TNFα significantly stimulated cancer epithelial migration. Similar to the monocyte recruitment, we also analyzed whether MCP1/CCL2 stimulated the MCF-7 cell migration. Our results indicate that the presence of a specific anti-CCL2 blocking antibody (in contact with migratory epithelial cells only during the time of assay) totally inhibits the epithelial migration stimulus of conditioned media from TNFα-pretreated preadipocytes (Figure 5B).

### 3.6. TNFα-Treated Preadipocytes Produce Snail Expression and Mitochondrial ROS That Stimulate Cancer Epithelial Migration

**Figure 6:**
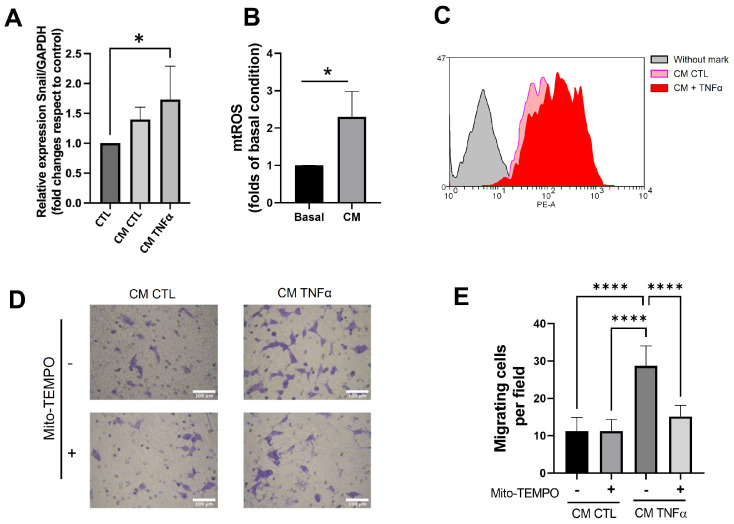
Conditioned media produced by TNFα-treated preadipocytes enhances snail gene expression and generates mitochondrial ROS that stimulate epithelial migration. (**A**) Snail mRNA expression of MCF-7 cells treated during 24 h with 50% conditioned media (CM) with preadipocytes derived from three different donors. (**B**) Mitochondrial ROS level of MCF-7 cells treated during 24 h with 50% conditioned media (MC) with preadipocytes derived from three different donors. Basal represents the production of ROS for MCF-7 cells treated (24 h) with CM from no-treatment preadipocytes. MC corresponds to the production of ROS by MCF-7 cells stimulated by TNFα-treated preadipocytes. (**C**) Representative histogram of the enhancement of mitochondrial ROS production induced by conditioned media derived from TNFα-pretreated preadipocytes. (**D**,**E**) MCF-7 cells were stimulated to migrate under the same conditions as in Figure 5B. Some stromal cells treated or not treated with TNFα (72 h) were also incubated (at the same time) with 1 µM mitoTEMPO. Afterwards, preadipocytes were cultured in serum-free media to condition media. Bars represent mean ± SD of migrating cells per field. Friedman test, followed by Bonferroni’s multiple comparisons test, were used to derive all *p* values. * *p* < 0.05 and **** *p* < 0.0001. Data correspond to three independent experiments performed with CM from three different samples.

Since the pro-inflammatory milieu produces epithelial-mesenchymal transition (EMT) and mitochondrial dysfunction in several models [25], we assessed whether the CM derived from TNFα-treated human mammary preadipocytes changed the snail gene expression, an early EMT marker, and mitochondrial ROS (mtROS) levels in MCF-7 cancer epithelial cells. TNFα-treated preadipocytes increased the snail expression (Figure 6A) and stimulated the mtROS production in MCF-7 cells compared to the CM of preadipocytes in the absence of TNFα (Figure 6B,C). To assess whether the production of ROS plays a role in MCF-7 migration, we treated preadipocytes with 1 µM mitoTEMPO, a mitochondria-targeted antioxidant, before these cells conditioned media (24 h) in serum-free media. Figure 6D shows that mitoTEMPO inhibited the capacity of conditioned media with TNFα-treated preadipocytes to stimulate epithelial migration, emphasizing the importance of mtROS production in the stimulus of migratory potential in cancer epithelial cells.

## 4. Discussion

In breast cancer, adipose cells are the more abundant component of breast stroma, establishing an inflammatory environment that recruits migratory cells and promotes tumorigenesis [26,27]. Moreover, the abundance of fat tissue, which is a characteristic of obese women, results in more aggressive tumors and worse prognostics [28]. In the present work, we described that TNFα inhibits mammary preadipocyte cell differentiation and promotes an inflammatory phenotype which mimics the tumor environment [29] and malignant characteristics through an increase in mtROS production.

As a result of the variety of forms of inflammation, the tumor microenvironment contains innate immune cells, with macrophages being the most abundant phenotype present. The abundance of these infiltrated cells (also named TAMs, for tumor-associated macrophages) generally correlates with a poor prognosis [30]. In line with this, we found that the conditioned media with TNFα-treated mammary pre-adipocytes stimulates the THP-1 monocyte recruitment.

Previous work recognized programmed death-ligand 1 (PD-L1), a molecule expressed by a variety of cells present in the tumoral microenvironment, as a critical immune checkpoint protein responsible for tumor immune evasion by inducing T cell exhaustion [31]. Interestingly, it has been identified that white adipocytes represent an essential source of PD-L1 signals, acting as an adipose checkpoint that regulates obesity-associated macrophage recruitment into adipose tissue, chronic inflammation, and metabolic dysfunction [32]. Moreover, it has been described that TNFα and IL-6, which are secreted by adipocytes, upregulate PD-L1 in hepatoma and B16-F1 cells, which may be at least partially involved in the role of obesity in promoting tumor progression [33]. The specific mechanism that mediates these activities is currently a matter of intense research.

Our results suggest that TNFα treatment significantly increases the production of MCP1/CCL2, an inflammatory marker, in mammary preadipocytes. Interestingly, preadipocytes have a significantly higher potential for producing MCP1/CCL2 compared with mature cells (Figure 3). Since an MCP1/CCL2-blocking antibody prevented the mobilization of either monocytes or epithelial MCF7 cells exposed to CM derived from TNFα-treated preadipocytes, the MCP1/CCL2 production by these stromal cells constitutes a reinforcement of the role of an inflammatory microenvironment in breast carcinogenesis [24].

Notably, TNFα alters the mitochondrial bioenergetics of several cell types [14,34,35,36,37]. It produces a rapid decrease in mitochondrial respiration [34] or an uncoupling of oxidative phosphorylation (OXPHOS), promoting the Complex I- and III-dependent mtROS production [35] or reverse electron transport [38]. In breast cancer cells, TNFα increases the glycolysis, lactate export, and expression of the glucose transporter 1 (GLUT1) and reduces the mitochondrial mass [14], offering an advantage adaptive to nutrient deprivation [14]. In TNFα-treated 3T3-L1 adipocytes, increased proton-leak-driven respiration decreased the mitochondrial membrane potential, and ATP turnover and mitochondrial fragmentation were reported [36,37]. Moreover, TNFα increased superoxide anion production and protein carbonylation in these cells [38].

Consistent with this, we observed that TNFα-treated preadipocyte-conditioned media exhibited increased snail expression, mtROS, and the migratory capacity of cancer epithelial cells. This latter event was dependent on the secretion of mtROS and MCP1/CCL2 by preadipocytes and seems also to depend on the establishment of a burst of mtROS in cancer epithelial cells, which may be triggered by MCP1/CCL2 and other soluble factors. The pro-inflammatory stimulation from TNFα promotes MCP1/CCL2 signaling [39], which is a mediator of metabolic adaptations in cells [40] and induces increased glycolysis in breast cancer cells [41].

The way in which this stromal, oxidative stimulus cooperates with other sets of signals to stimulate epithelial motility is a matter of our present interest. We believe that the study of the close association between inflammation and breast cancer metabolism will provide new strategies to fight the progression of the disease in the future.

## 5. Conclusions

The present work described the inhibitor effect of TNFα on human mammary preadipocyte differentiation and its role in establishing a proinflammatory microenvironment that favors monocyte recruitment and mammary epithelial migration. We also described that these TNFα-induced activities are contingent upon the stromal production of MCP1/CCL2 and an increase in epithelial mitochondrial ROS.

## Figures and Tables

**Table 1:** Primer list used for qPCR.

Accession Number	Target mRNA	Forward Primer (5′⟶3′)	Reverse Primer (5′⟶3′)
NM_000576.3	IL1-β	AATCCCCAGCCCTTTTGTTG	AAATGTGGCCGTGGTTTCTG
NM_002982.4	CCL2	TGTCCCAAAGAAGCTGTGATCT	GGAATCCTGAACCCACTTCTG
NM_138712.5	PPARγ	TTCCCGCTGACCAAAGCAAA	ACTGGCAGCCCTGAAAGATG
NM_001442.3	aP2	TGCAGCTTCCTTCTCACCTTGA	TCCTGGCCCAGTATGAAGGAAATC
NM_002046.7	GAPDH	TTGCCATCAATGACCCCTTC	TGATGACAAGCTTCCCGTTC

## Data Availability

All of the data is contained within the article and the supplementary materials.

## Supplementary Materials

The following supporting information can be downloaded at: https://www.mdpi.com/article/10.3390/antiox12040813/s1, Figure S1: TNFα stimulates the expression of MCP1/CCL2 in differentiated mammary adipocytes.
