# Supplementary material for: TNF-Alpha Promotes an Inflammatory Mammary Microenvironment That Favors Macrophage and Epithelial Migration in a CCL2- and Mitochondrial-ROS-Dependent Manner
Source: Antioxidants (Basel). 2023 Mar 27;12(4):813. doi: 10.3390/antiox12040813 (PMC10135343; doi:10.3390/antiox12040813)
Supplement: Supplementary file 1 [file antioxidants-12-00813-s001.zip › antioxidants-2244140-supplementary.pdf]

TNF-alpha promotes an inflammatory mammary microenvironment that favors macrophage and epithelial migration in a CCL2 and mitochondrial ROS-dependent manner.

María Jesús Vera [1,4], Francisco Guajardo [2,3,4], Felix A. Urra [2,3,4], Nicolás Tobar [1*] and Jorge Martínez [1,4,*]

Supplementary Figure S1.

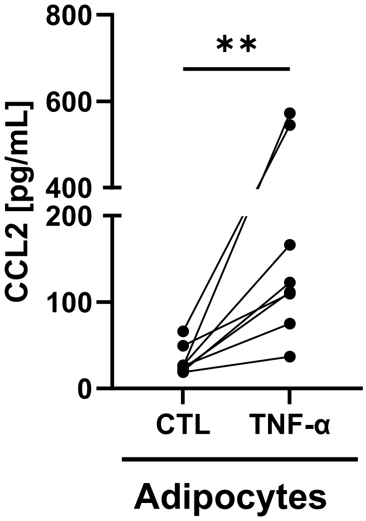

Figure S1. TNFα stimulates the expression of CCL2 in differentiated mammary adipocytes. Preadipocytes derived from mammary tissue from eight healthy patients were differentiated for 14 days and treated with TNFα (20 ng/ml) for 72 h. Then differentiated mammary adipocytes was allowing to conditioned media for 24 hours. Expression of MCP1/CCL2 was analyzed using an ELISA assay as indicated in material and method. Data correspond to the mean ± SD. Paired Wilcoxon test was used to deriving p values. (**) corresponding to $p<0.01$.
